# Alterations of ocular surface microbiome in glaucoma and its association with dry eye

**DOI:** 10.1099/jmm.0.002013

**Published:** 2025-05-13

**Authors:** Houyem Kamdougha, Bernard Taminiau, Papa Abdoulaye Fall, Saloua Ben Amor, Amira Trigui, Georges Daube, Basma Mnif

**Affiliations:** 1 Laboratory of Microbiology, Habib Bourguiba University Hospital, Sfax University, Sfax, Tunisia; 2 Laboratory of Microbiology, Department of Food Sciences, Fundamental and Applied Research for Animal & Health (FARAH), Faculty of Veterinary Medicine, University of Liège, 4000 Liège, Belgium; 3 Department of Ophthalmology, Habib Bourguiba University Hospital, University of Sfax, Sfax, Tunisia; 4 Research Laboratory Microorganisms and Human Disease "MPH LR03SP03", Sfax University, Sfax, Tunisia

**Keywords:** conjunctiva, dry eye disease, dysbiosis, glaucoma, microbiome, preservatives

## Abstract

**Introduction.** Alterations in ocular surface microbiota (OSM) have been noted in both dry eye disease (DED) and glaucoma. However, the combined effects of these conditions on OSM have not been explored.

**Hypothesis.** We hypothesized that patients with both glaucoma and dry eye would exhibit distinct changes in OSM composition and diversity compared to those with only glaucoma, only dry eye or healthy individuals.

**Aim.** We employed amplicon sequencing to investigate OSM profiles in patients with glaucoma and/or dry eye disease.

**Methods.** Swab samples from the conjunctiva of both eyes were collected from 28 glaucomatous patients [13 without dry eye syndrome (G-only) and 15 with dry eye syndrome (G-DED)], 13 DED patients without glaucoma (DED-only) and 31 age-matched healthy controls (HCs). After V3–V4 16S rRNA sequencing, MOTHUR tools and R language were used to elucidate and compare OSM composition and diversity between groups.

**Results.** Our data revealed very diverse bacterial communities with 28 phyla and 785 genera. All the groups shared the three most abundant phyla, *Actinobacteria* (67.47%), *Firmicutes* (17.14%) and *Proteobacteria* (13.73%). *Corynebacterium* (54.75%), *Staphylococcus* (10.71%), *Cutibacterium* (8.77%) and *Streptococcus* (3.20%) were the most abundant genera. Only the G-DED group showed higher alpha diversity than the HC group (*P*<0.05). However, significant differences in beta diversity were observed between all three patient groups and the HC group. The Differential Expression for Sequencing 2 (DESeq2) analysis unveiled an increased presence of opportunistic bacteria across all pathological groups, with the G-DED group demonstrating the most pronounced alterations.

**Conclusions.** Our findings confirm the predominance of Gram-positive bacteria in normal OSM and the rise of opportunistic Gram-negative bacteria in glaucoma and dry eye disease. This is the first study to characterize OSM in glaucoma patients with DED.

## Introduction

Glaucoma is a progressive optic neuropathy marked by the loss of peripapillary retinal nerve fibres, apoptosis of ganglion cells and a gradual decline in the visual field. Worldwide projections indicate that 64.3 million individuals aged 40–80 years were affected by glaucoma in 2019, with this number rising to 76.0 million by 2020 and expected to reach 111.8 million by 2040 [1]. Although glaucoma is the second leading cause of blindness globally [2], its underlying mechanisms remain not yet fully clarified [3]. Risk factors for glaucoma encompass advancing age, gender, dietary habits, obesity, anxiety [4] and elevated intraocular pressure (IOP), which is the primary modifiable risk factor for glaucoma [2]. Consequently, the primary therapeutic approach for glaucoma typically involves the use of lowering IOP eye drops. As these eye drops commonly contain active ingredients and preservatives, repeated exposure to them may increase the likelihood of epithelial damage, inflammation and discomforting symptoms, such as tear film layer instability, a burning sensation and dryness [2,5, 6].

Recent studies have emphasized a significant overlap between glaucoma and dry eye disease (DED), with DED being notably more prevalent among individuals with glaucoma than in the general population [2,7,9]. The estimated prevalence of DED in people with glaucoma varies between studies [10,14]. For instance, Erb *et al*. found that 52.6% of glaucoma patients were diagnosed with DED [11], while Monjane and Makupa reported a DED prevalence of 79.7% among glaucoma patients in Tanzania [7]. This high DED prevalence underscores the importance of understanding the interplay between these two conditions, which is complex and multidimensional [15].

Glaucoma treatments, particularly those containing preservatives, can substantially alter the ocular surface environment, promote inflammation and disrupt the balance of the tear film [9,11, 16,23]. Reciprocally, these persistent inflammatory changes may not only exacerbate DED symptoms but also impact the effectiveness of glaucoma treatment, reduce patient adherence to therapy and diminish overall quality of life [9,15].

Advancements in DNA sequencing have brought increased attention to site-specific microbiomes, including the ocular surface microbiota (OSM) and its crucial role in health and disease [24,26]. While OSM dysbiosis has been implicated in the development of ophthalmic diseases, including DED [27], its involvement in glaucoma remains poorly explored. Some studies suggest that glaucoma patients using antiglaucoma drops, especially those preserved with benzalkonium chloride (BAC), exhibit altered OSM compositions, including an increased presence of Gram-negative bacteria and antibiotic-resistant strains [6,28,30]. Similar microbiome alterations have been observed in DED patients [31,34], further reinforcing the possible interplay between microbiota changes, ocular surface inflammation and glaucoma progression [34,38].

However, the interplay between glaucoma, DED and OSM perturbation has not yet been studied. Therefore, we aimed to investigate the association between the conjunctival microbiota and glaucoma with or without dry eye syndrome by analysing the conjunctival microbiota of a cohort of Tunisian participants.

This study holds significant implications for clinical care. A deeper understanding of the association between glaucoma, DED and OSM perturbations could inform innovative management strategies that address both disease mechanisms and their impact on the ocular surface. Furthermore, this study opens the door for the development of new glaucoma medications or regimens designed to minimize adverse effects on the ocular surface. Understanding how glaucoma therapies influence the OSM and contribute to DED underscores the need for an integrated approach to glaucoma care – one that prioritizes both effective IOP control and the preservation of ocular surface health. Ultimately, this exploration bridges a critical knowledge gap, offering insights with the potential to transform patient management and improve quality of life.

## Methods

### Study design

All participants enrolled were over 40 years old and were recruited within the period from May 2021 to May 2023. The patients were enrolled from the Ophthalmology ward, Habib Bourguiba University Hospital, Sfax, Tunisia. The study involved a total of 72 participants, comprising 31 healthy controls and 41 patients. Among the patients, 13 had only glaucoma, 13 had only DED and 15 had both glaucoma and dry eye syndrome. We were unable to achieve a balanced gender distribution due to the higher prevalence of DED among females.

Healthy subjects were defined as individuals without any history of ocular disease. Exclusion criteria included the use of artificial tears and local or systemic antibiotics in the last 4 weeks, the use of contact lenses during the same period, a history of ocular and periocular infection within 3 months before enrolment and a history of ocular surgery in the last 3 months. The 4-week exclusion period for artificial tears and antibiotics allows the restoration of the natural OSM and reduces the treatment confounding effects on the OSM.

### Sample collection

Before sample collection, a drop of oxybuprocaine chlorhydrate from a new unopened bottle was administered to each eye for 3 min. Subsequently, a sterile flocked nylon swab (FLOQSwabs #518CS01, Copan, Italy) was swiped three times over the lower conjunctival fornix without touching the eyelids by the same ophthalmologist clinician. Conjunctival swabs were collected from both eyes of each subject. All samples were promptly stored at −80 °C until analysis. For the negative control, samples from the used bottle were taken before and after conjunctival collection and processed in parallel with the participant samples.

### Quantitative PCR

Total bacterial DNA was extracted using the QIAamp PowerFecal Pro DNA Kit, following the manufacturer’s recommendations. Quantitative PCR (qPCR) was performed to estimate the bacterial load in the sample using the Applied Biosystems (Foster City, CA, USA) 7500 Fast Real-Time PCR system. For total bacteria, the quantification by qPCR of V1–V2 region of 16S rRNA gene was conducted after the addition of an SYBR Green fluorescent molecule in the reaction mix using 10 µM each of forward (5′-ACT-CCT-ACG-GGA-GGC-AGC-AG-3′) and reverse (5′-ATT-ACC-GCG-GCT-GCT-GG-3′) primers [39].

### 16S ribosomal DNA amplicon sequencing

The V1–V3 region of the 16S ribosomal DNA (rDNA) was amplified by PCR, and the library preparation was conducted using the following primers: forward (5′- GAGAGTTTGATYMTGGCTCAG-3′) and reverse (5′-ACCGCGGCTGCTGGCAC-3′). Subsequently, the purification of each PCR product was performed using the Agencourt AMPure XP Ball Kit (Beckman Coulter, Pasadena, CA, USA), followed by a second round of PCR for indexing using Nextera XT index primers 1 and 2. After purification, the quantification of the PCR products was carried out using the Quant-IT PicoGreen (ThermoFisher Scientific, Waltham, MA, USA) and diluted to a concentration of 10 ng µl^−1^. A final qPCR quantification of each library sample was conducted using the KAPA SYBR® FAST qPCR Kit (Kapa Biosystems, Wilmington, MA, USA) before normalization, pooling and sequencing on a MiSeq sequencer by v3 reagents (ILLUMINA, Illumina Netherlands, Eindhoven, The Netherlands) [39].

### Microbiota profiling

The raw sequences were assembled by FLASH (v1.2.7). Then, sequence read processing was performed using, respectively, the MOTHUR v1.47 package and VSearch algorithm [40] for alignment, clustering and chimaera detection [41].

Following the cleaning process, sequences were clustered into operational taxonomic units (OTUs) at 97% of identity. 16S rDNA reference alignment and taxonomical identification relied on the silva database (v1.38.1) of full-length 16S rDNA sequences. Subsequently, a rarefied table consisting of 10,000 reads per sample was employed for subsequent analysis. The reads were then aggregated into phylotypes at various taxonomic levels, including phylum, class, order, family, genus and species.

### Biostatistical analyses

The alpha diversity metrics were assessed using the Shannon diversity index, the Simpson index and the Chao1 index with the vegan R package [42] and tested by the ANOVA. Then, the Bonferroni method was used to correct the results of ANOVA.

The bacterial beta diversity was compared using dissimilarity matrices, based on the Bray–Curtis index (composition) or Jaccard index (structure), and the results were visualized in a non-metric multidimensional scaling model (NMDS) plot with vegan3d and rgl R package. Then, the pairwise.adonis2 function from the pairwise.adonis R package was employed for conducting permutational ANOVA, and pairwise comparisons were conducted using the pairwise Adonis function from the pairwise Adonis R package. These statistical tests were applied to assess beta diversity in the analysis. Bacterial profiles were visualized by stacked bar chart with tidyr R package.

DESeq2 was used to identify differentially abundant phyla and genera between groups in raw count data (significant genera defined as log2 fold change >2 and *P*-adjusted values<0.05). Then, the DESeq2 results were visualized by a volcano plot with ggplot R package. The comparison between paired eye samples was performed using the Bray–Curtis dissimilarity and visualized by the dendrogram using the FigTree v1.4.4 application. Metagenome inference was conducted using the Phylogenetic Investigation of Communities by Reconstruction of Unobserved States (PICRUST2).

All data and statistical analyses were conducted using R Studio (v4.3.1).

Statistical significance was defined at a threshold of *P*-values<0.05 [43].

## Results

### Subject characteristics

The demographic details of 72 participants of each group are presented in Table 1. All pathological groups were carefully matched for age with the healthy control group (*P*>0.05). However, the mean age was significantly higher in the G-only group than in the DED-only group (*P*<0.05) (ANOVA, adjusted using the Bonferroni method). The women were also significantly overrepresented in the G-DED and DED-only groups (*P*>0.05) (Pearson’s chi-squared test). The clinical characteristics of patients of each group are noted in Tables 2 and S1 (available in the online Supplementary Material) (File S1).

**Table 1.** Participant demographics: gender and age distribution among G-only, G-DED, DE-only and HC groups

	HC	G-only	G-DED	DED-only	*P*-value
**Sample size (** ***n*** **)**	31	13	15	13	na
**Age (years,mean±** **sd** **)**	64.58±9.89	69.54±7.85	63.13±9.48	59.92±12.15	0.2 (HC vs G-only)0.86 (HC vs G-DED)0.17 (HC vs DED-only)0.088 (G-only vs G-DED)0.005** (G-only vs DED-only)0.65 (G-DED vs DED-only)
**Sex (** ***n*** **, %female/male)**	12/19 (38.7/61.29)	4/9 (30.76/69.23)	11/4 (73.33/26.66)	9/4 (69.23/30.76)	0.68 (HC vs G-only)0.0022** (HC vs G-DED)0.006** (HC vs DED-only)0.003** (G-only vs G-DED)0.003** (G-only vs DED-only)1 (G-DED vs DE-only)
**Left eyes** (number)	31	13	15	13	na
**Right eyes** (number)	29	11	15	12	na

*P<0.05; **P<0.01; ***P<0.001 *(*ANOVA followed by the post hoc Tukey test and Pearson’s chi-squared test).

**Table 2.** Clinical characteristics of patients (G-only, G-DED and DED-only)

Patient	Treatment	Preservative	Diabetes	Hypertension
**G-only1**	Brimonidine, latanoprost, hypromellose	BAC	−	−
**G-only2**	Timolol, dorzolamide, latanoprost	BAC	−	−
**G-only3**	Latanoprost	BAC	−	−
**G-only4**	Travoprost, Carteolol hydrochloride	Polyquaternium-1+BAC	−	−
**G-only5**	Dorzolamide, timolol, brimonidine, latanoprost	BAC	−	−
**G-only6**	Bimatoprost, dorzolamide/timolol	BAC	−	−
**G-only7**	Carteolol hydrochloride and dorzolamide	BAC	+	+
**G-only8**	Dorzolamide, latanoprost	BAC	+	+
**G-only9**	Dorzolamide, latanoprost	BAC	+	+
**G-only10**	Latanoprost, dorzolamide/timolol	BAC	−	−
**G-only11**	Carbomer 980, timolol, latanoprost	Cetrimide+BAC	+	−
**G-only12**	Timolol, dorzolamide, latanoprost	BAC	−	−
**G-only13**	−	−	−	−
**G-DED1**	Timolol, bimatoprost, carbomer 980	BAC	−	−
**G-DED2**	Carbomer 980, latanoprost	Cetrimide+BAC	−	−
**G-DED3**	Latanoprost, carbomer 980, brinzolamide, carbomer 974P	Cetrimide+BAC	−	−
**G-DED4**	Timolol, dorzolamide, latanoprost	BAC	−	−
**G-DED5**	Timolol, dorzolamide, brimonidine, carbomer 980	Cetrimide+BAC	+	−
**G-DED6**	Timolol, dorzolamide, brimonidine, carbomer 980	Cetrimide+BAC	−	+
**G-DED7**	Brimonidine, hypromellose, carbomer 974P	BAC	−	−
**G-DED8**	Timolol, latanoprost, sodium carmellose	BAC	−	−
**G-DED9**	Chlorhydrate de carteolol, carbomer 974P, brimonidine	BAC	+	+
**G-DED10**	Travoprost, dorzolamide, hypromellose, carbomer 980	Polyquaternium-1+cetrimide+BAC	−	−
**G-DED11**	Latanoprost, hypromellose, carbomer 974P	BAC	−	−
**G-DED12**	Dorzolamide/timolol, travoprost, carbomer 974P	Polyquaternium-1+BAC	−	−
**G-DED13**	Timolol, dorzolamide, carbomer 974P	BAC	−	−
**G-DED14**	Timolol, dorzolamide, carbomer 980	Cetrimide+BAC	−	−
**G-DED15**	Travoprost, timolol (stopped treatment for 3 months)	Polyquaternium-1+BAC	+	−
**DED-only 1**	Untreated	−	−	−
**DED-only 2**	Indometacin	−	+	−
**DED-only 3**	Hypromellose, carbomer 980	Cetrimide	−	−
**DED-only 4**	Hypromellose, carbomer 980	Cetrimide	−	−
**DED-only 5**	Povidone	−	−	−
**DED-only 6**	Thealose	−	−	−
**DED-only 7**	Hypromellose	−	−	−
**DED-only 8**	Carbomer 980	−	−	−
**DED-only 9**	Carbomer 980, hypromellose, sodium hyaluronate	−	−	−
**DED-only 10**	Hypromellose	−	−	−
**DED-only 11**	Hypromellose, carbomer 980	Cetrimide	−	−
**DED-only 12**	Povidone (stopped treatment for 2 months)	BAC	−	−
**DED-only 13**	Carbomer 974P	BAC	−	−

−, absence; +, presence.

In total, 144 eye samples were obtained from the 72 participants, of which 5 samples did not provide enough usable sequences to be included in the analysis and were excluded. Similarly, the negative controls did not generate sufficient exploitable sequences, further confirming the reliability of the results. After the removal of non-conforming sequences and standardizing the number of sequences per sample, the 139 samples were characterized by 1,390,000 high-quality sequences, with a depth of 10,000 sequences per sample. The sequences are accessible via BioProject ID (PRJNA1145861) in the NCBI database.

#### Conjunctival microbiota associated with pathology

##### Total 16S rDNA copy quantification (qPCR)

**Fig. 1.**
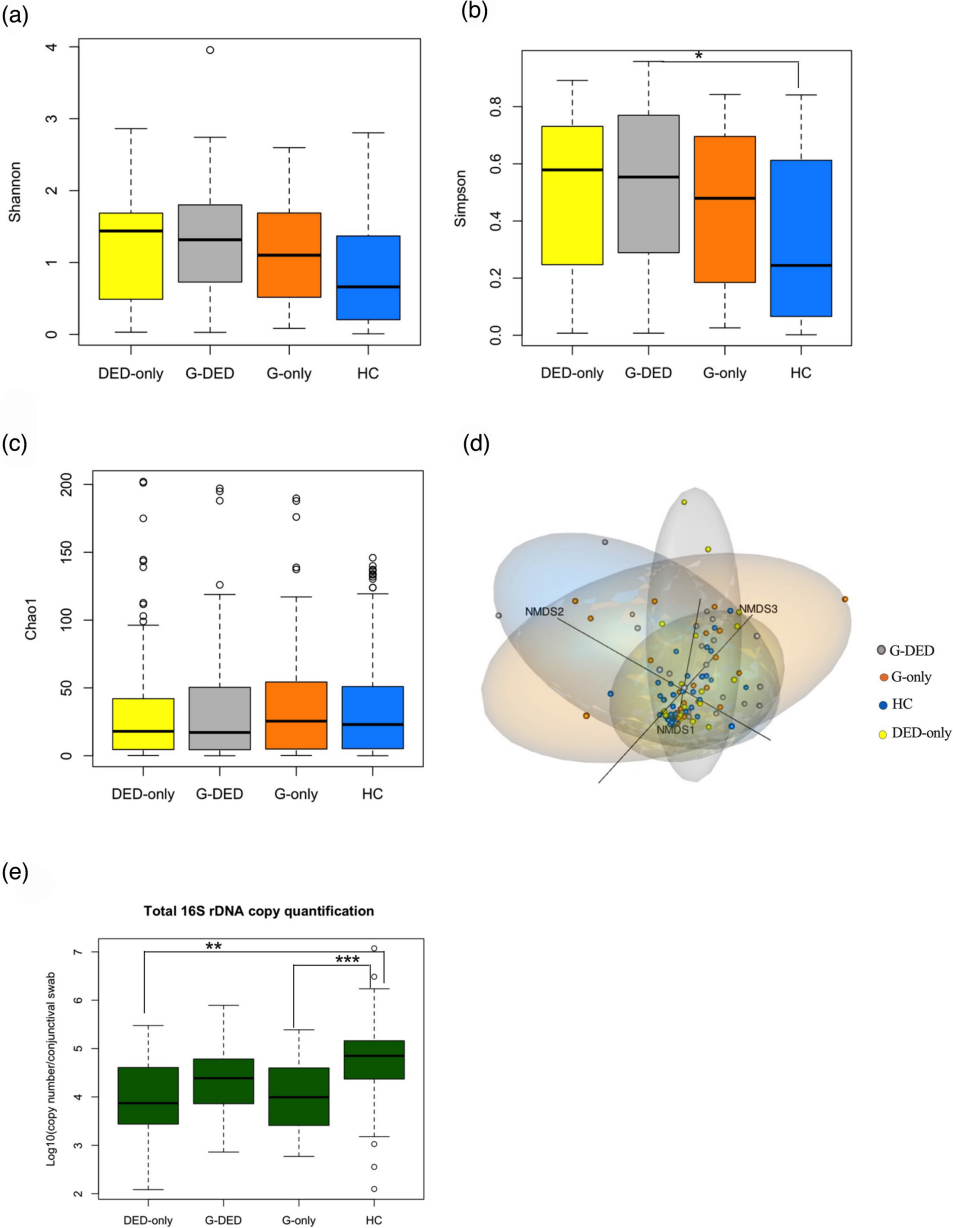
(a–c) Boxplot visualization of alpha diversity (based, respectively, on the Shannon index and Simpson index) and richness (based on the Chao1 index) of the four study groups, tested by ANOVA, followed by paired t-tests with the *P*-value corrected by Bonferroni (threshold value of *P*=0.05). (d) NMDS model based on a Bray–Curtis dissimilarity matrix, with data projected into three-dimensional space (*K*=3) and a model stress of 0.1028451. The ellipses drawn around the groups show the 95% confidence intervals around the centroid of each group, illustrating the dispersion and concentration of the samples. (e) Boxplot visualizing the quantification of total bacterial 16S rDNA. The difference in bacterial load is tested by ANOVA, followed by paired t-tests with the *P*-value corrected by Bonferroni (threshold value of *P*=0.05). DED-only, dry eye group; G-only, glaucoma group; G-DED, glaucoma with dry eye group.

In healthy conjunctival microbiota, the mean copy number of total 16S rDNA was 10^e^ (4.76±0.86) copy number/conjunctival swab. The 16S rDNA copy number of conjunctival bacteria in the healthy group samples was significantly higher than that recorded in the G-only and DED-only groups (*P*<0.005, ANOVA test adjusted using the Bonferroni method) (Fig. 1, Table 3).

**Table 3.** Comparison of alpha diversity, beta diversity and qPCR between the different groups

Group	Shannon	Simpson	Chao1	Bray–Curtis method	Jaccard method	Log10 (qPCR)
				*P*-value	*P*-value	*P*-value
G-only vs HC	1	0.41	1	0.001***	0.001***	0.0002***
G-DED vs HC	0.05	0.02*	0.48	0.02*	0.011*	0.1957
DED-only vs HC	0.30	0.15	0.16	0.013*	0.011*	0.0013**
G-only vs DED-only	1	1	0.48	0.228	0.211	1
G-only vs G-DED	1	1	1	0.097	0.084	0.63063
DED-only vs G-DED	1	1	1	0.934	0.909	0.77059

*P<0.05; **P<0.01; ***P<0.001 (ANOVA, adonis2).

##### Alpha diversity

The ANOVA test across all metrics indicated a significant difference in the alpha diversity by Simpson index between the DED-only group and healthy controls (*P*-value <0.05 ANOVA, corrected by Bonferroni) (Fig. 1, Table 3).

##### Beta diversity

The adonis2 showed a statistically significant difference in the beta diversity between the healthy group and the three pathological groups (*P*<0.05; permutational multivariate analysis pairwise.adonis2) (Fig. 1, Table 3)

Differences in diversity indices and PCR quantification are evaluated using an ANOVA test, followed by paired t-tests with *P*-values corrected by Bonferroni (threshold value of *P*=0.05). Differences between groups for beta diversity are assessed using an adonis2 test with 1,000 permutations, followed by pairwise tests (pairwise.adonis) with 1,000 permutations and *P*-values corrected using Bonferroni (threshold value of *P*=0.05).

### Taxonomic composition and dominant genera and phyla

The taxonomic assignment of bacterial reads and their hierarchical classification revealed 28 phyla and 785 genera in conjunctival swabs.

**Fig. 2.**
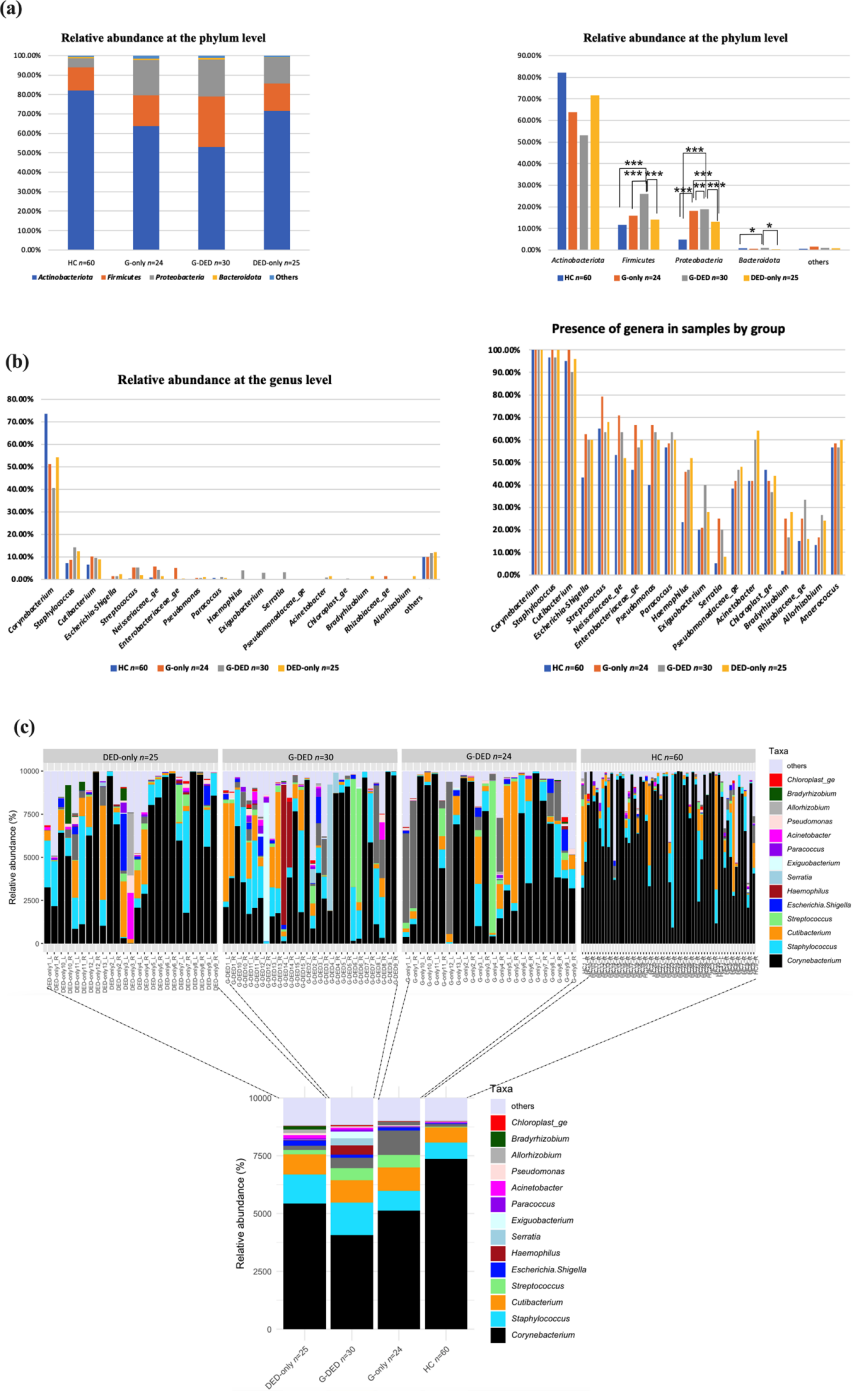
Taxonomy bar plot of microbial composition in the control (HC) and pathological groups [glaucomatous patients (G-only), glaucomatous patient with DED (G-DED) and dry eye syndrome group (DED-only)] eye samples. The most 18 abundant genera were represented in the figure. The relative abundance for each individual sample was initially computed by dividing the read counts assigned to each OTU linked to each microbe by the total read count of the sample. (**a**) Composition of the ocular microbiome at the phylum level with a comparison of phylum abundance between groups using DESeq2. The two groups G-DED and G-only contained a significantly higher abundance of *Proteobacteria* compared to the control and the DED-only groups (*P*<0.05). The G-DED group contained a significantly higher abundance of *Firmicutes* in contrast to the other three groups (*P*<0.05) and a significantly higher abundance of *Bacteroidota* in comparison with the HC and DED-only groups. (**b, c**) Composition of sample eye microbiota based on relative abundance at the genus level per sample and per group. The top six abundant genera in almost all groups were *Corynebacterium*, *Staphylococcus*, *Cutibacterium, Escherichia-Shigella*, *Streptococcus* and *Neisseriaceae_ge*.

For all groups at the phylum level, the three dominating bacteria, accounting for more than 98% of the total abundance, were *Actinobacteria* (67.47%), *Firmicutes* (17.14%) and *Proteobacteria* (13.73%). The most abundant phylum was *Actinobacteria* in all four groups, with median relative abundances at 82.21% among healthy controls, 63.77% in the glaucoma group, 53.04% in the glaucoma with dry eye group and 71.63% in the dry eye group. The DESeq2 test showed that the *Firmicutes* were significantly enriched in the G-DED group (26.02%, *P*<0.0001) compared to the three other groups [G-only (15.94%), DED-only (14.13%) and HC (11.69%)]. *Proteobacteria* were significantly higher in both the glaucoma group (18.14%, *P*<0.001) and G-DED group (18.92%, *P*<0.005) in contrast to the DED-only group (13.12%) and healthy group (4.74%). Despite its low abundance, the *Bacteroidota* was significantly higher in the G-DED group in comparison with the HC and DED-only groups (0.97%, *P*<0.005). At the genus level, the top seven abundant genera and their relative abundance in the HC, G-only, G-DED and DED-only groups were as follows, respectively: *Corynebacterium* (73.59, 51.22, 40.67 and 54.29%), *Staphylococcus* (7.07, 8.59, 14.14 and 12.55%), *Cutibacterium* (former name *Propionibacterium*) (6.60, 10.19, 9.56 and 8.74%), *Escherichia-Shigella* (0.21, 1.39, 1.45 and 2.29%), *Streptococcus* (0.39, 5.25, 5.27 and 1.90%), *Neisseriaceae_ge* (0.85, 5.57, 4.24 and 1.41%) and *Paracoccus* (0.47, 0.21, 0.87 and 0.57%). Except for *Corynebacterium,* all these genera were significantly higher in the G-DE group in comparison to the healthy group (*P*<0.05). In addition, *Staphylococcus*, *Cutibacterium*, *Streptococcus* and *Escherichia-Shigella* were significantly enriched in the DED-only group compared with the healthy group (*P*<0.05), and *Cutibacterium*, *Streptococcus*, *Neisseriaceae_ge* and *Escherichia-Shigella* were significantly more abundant in the G-only group in contrast to the healthy group (*P*<0.05) [Table S2 (File S1) and Fig. 2].

### Minimal core OSM

We identified four genera (*Corynebacterium*, *Staphylococcus*, *Cutibacterium* and *Streptococcus*) present in more than 60% of participants. The three first genera were present in all G-only group samples (100%) and in more than 89% of all samples. Only *Corynebacterium* was present in all samples (100%) (Fig. 2).

### Differential abundance analysis

**Fig. 3.**
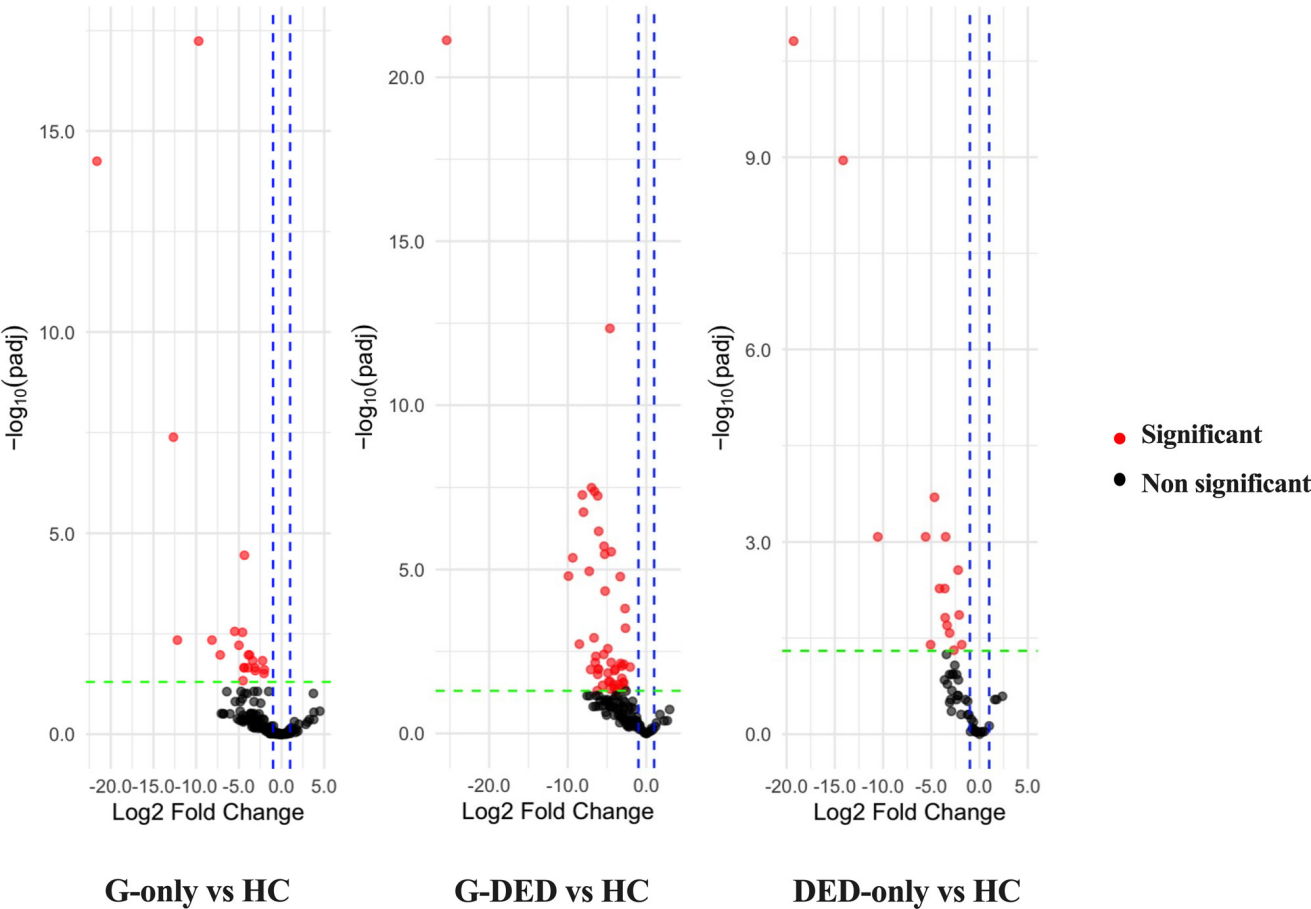
Marker genera volcano plots depict bacterial taxa and their log2 fold change, comparing eye samples from patients in the G-only group, G-DED group and DE-only group to those of healthy controls by identifying the adjusted *P*-value. The log2 fold change and padj values are derived from the DESeq2 workflow. Genera exhibiting mean count with a significance level of *P*<0.05 are marked as red points (indicating a significant difference between groups), while black points signify no significant difference.

DESeq2 revealed significant differences in the bacterial distribution between groups [Table S2 (File S1) and Fig. 3]. At the phylum level, we detected an increase in the abundance of *Firmicutes* in the G-DED group in comparison with other groups (*P*<0.0001) and an increase of *Proteobacteria* in both the G-DED and G-only groups in comparison with the healthy and DED-only groups (*P*<0.05). At the genus level, the most abundant genera significantly increased in the G-DED group vs healthy controls were *Staphylococcus*, *Streptococcus*, *Haemophilus*, *Cutibacterium*, *Serratia*, *Paracoccus*, *Bradyrhizobium*, *Pseudomonas*, *Escherichia-Shigella*, *Neisseriaceae_ge*, *Acinetobacter*, *Exiguobacterium* and *Kocuria* (*P*<0.05). A similar pattern was observed for the G-only group with the increased abundance of *Cutibacterium*, *Streptococcus*, *Pseudomonas*, *Escherichia-Shigella*, *Granulicatella*, *Enterobacter*, *Actinomyces*, *Bradyrhizobium*, *Proteus* and some unclassified genera from *Enterobacteriaceae* and *Corynebacteriaceae* (*P*<0.05). The DED-only group also showed a significant increase in some genera compared with the healthy group, including *Bradyrhizobium*, *Pseudomonas*, *Streptococcus*, *Cutibacterium*, *Staphylococcus*, *Acinetobacter*, *Escherichia-Shigella*, *Haemophilus*, *Veillonella* and unclassified *Enterobacteriaceae* (*P*<0.05).

#### Conjunctival microbiota profiling in the right and left eyes

**Fig. 4.**
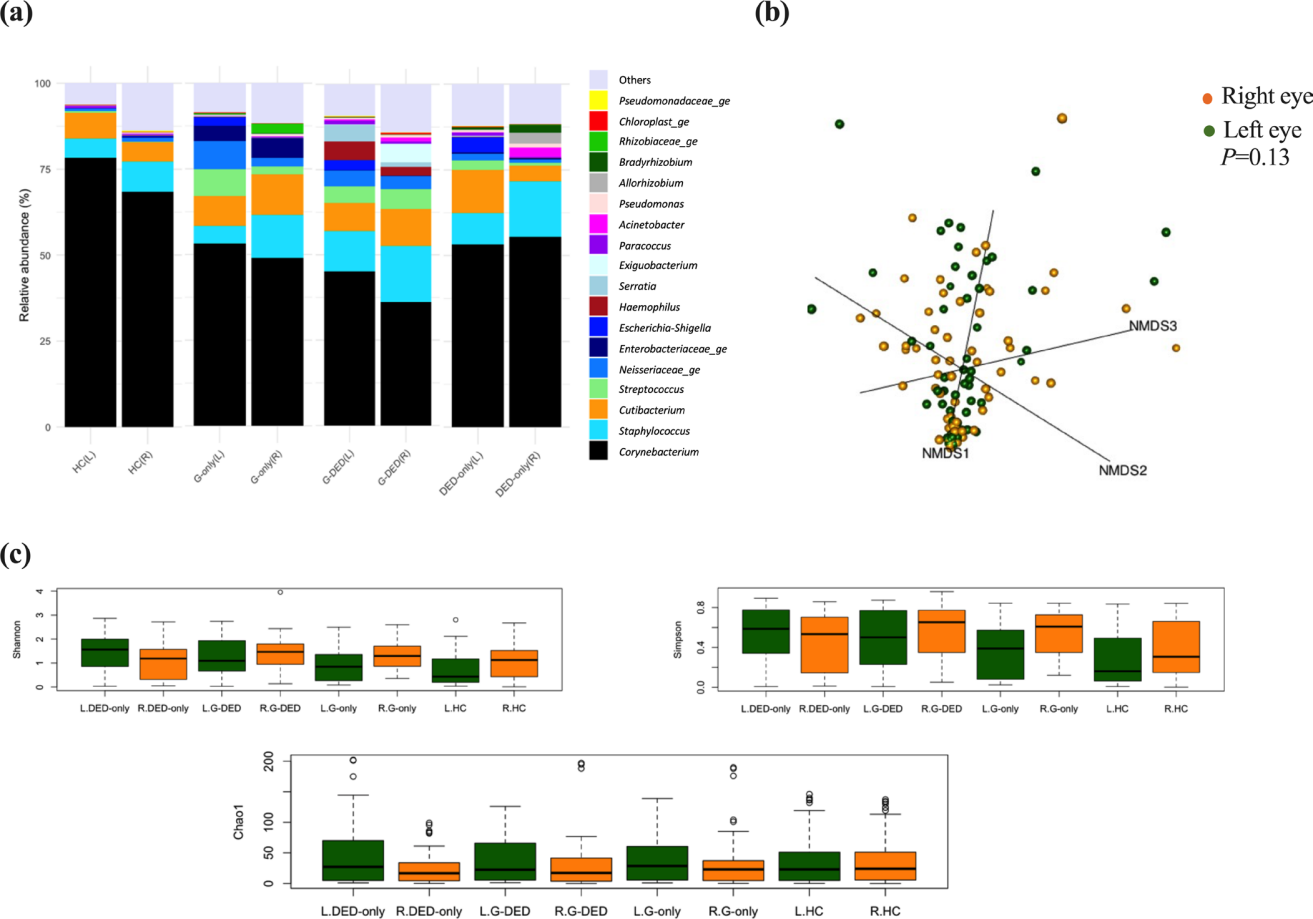
Stacked bar chart visualization of bacterial profiles of left and right eyes by group with ggplot from the R package (**a**). NMDS model based on a Bray–Curtis dissimilarity matrix, with data projected into three-dimensional space (*K*=3) and a model stress of 0.1028451 (**b**). Boxplot visualization of alpha diversity (based, respectively, on the Shannon index and Simpson index) and population richness (based on the Chao1 index) of the microbiota of left and right eyes, tested by Wilcoxon test (threshold value of *P*=0.05) (**c**). DED-only, dry eye group; G-only, glaucoma group; G-DED, glaucoma with dry eye group; L, left eye; R, right eye.

**Fig. 5.**
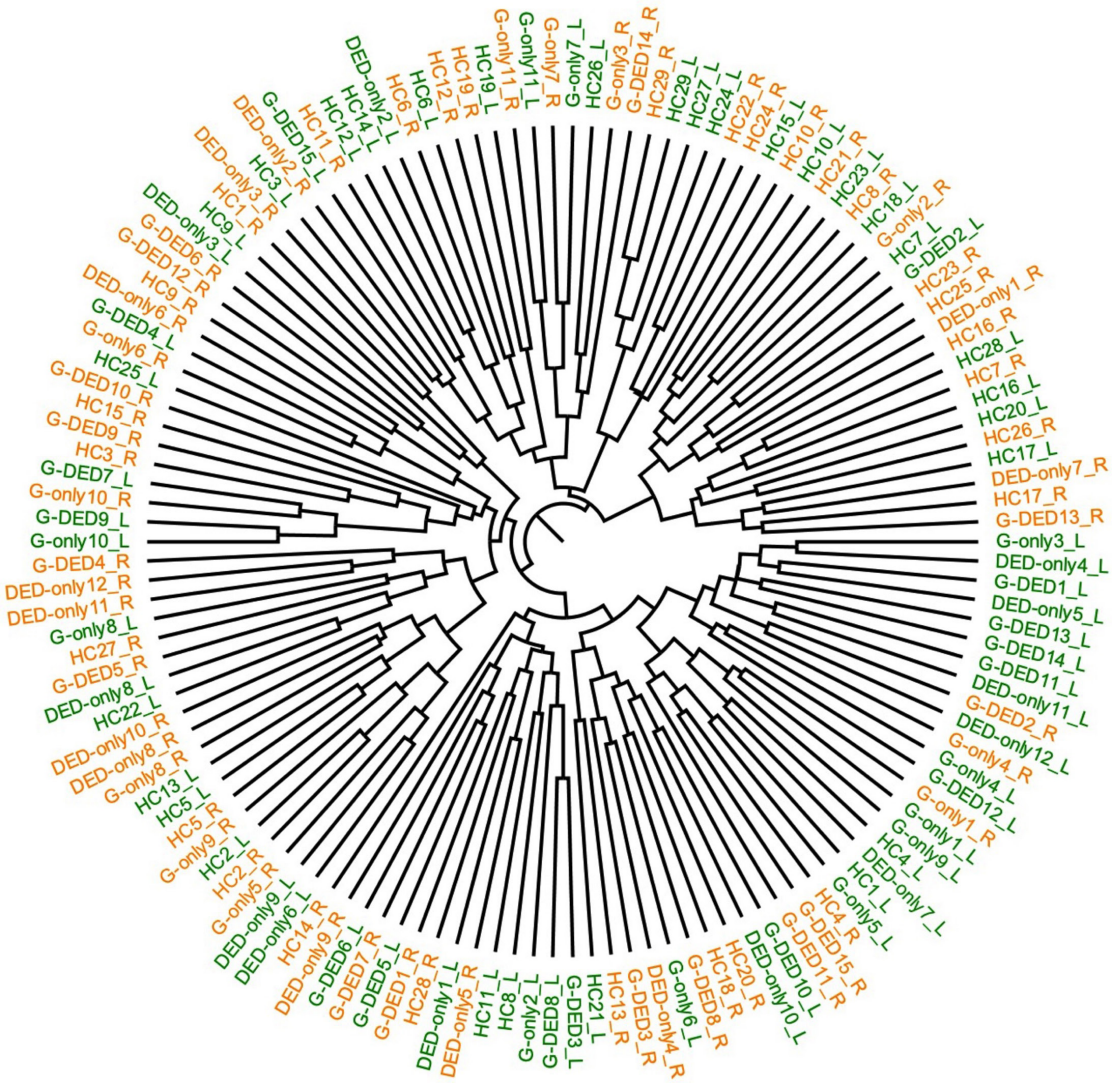
Bray–Curtis dendrogram of left and right eye microbiota of each participant based on the Bray–Curtis dissimilarity. >0.6, high dissimilarity score; <0.6, low dissimilarity score. DED-only, dry eye group; G-only, glaucoma group; G-DED, glaucoma with dry eye group; L, left eye; R, right eye.

Alpha diversity analysis based on Shannon, Simpson and Chao1 indexes did not detect any significant difference between left eyes and right eyes in all groups (*P*>0.05, Wilcoxon). Similarly, beta diversity analysis showed no significant difference between the two eye microbiota by group (*P*>0.05, adonis2) (Table 4, Fig. 4). However, the individual comparison, calculated by the Bray–Curtis dissimilarity (File S2) and visualized by the Bray–Curtis dendrogram (Fig. 5), showed a high dissimilarity score (>0.6) between the left and right eye of the same person in 59.7% of participants (40/67), either from patients or healthy participant (File S2).

**Table 4.** Comparison of alpha diversity and beta diversity of microbiota between left and right eyes by group

Group	Shannon	Simpson	Richness	Bray–Curtis	Jaccard
Comparison	*P*-value (Wilcoxon)	*P*-value (Wilcoxon)	*P*-value (Wilcoxon)	*P*-value (adonis2)	*P*-value (adonis2)
HC (R vs L)	0.15	0.11	0.68	0.63	0.68
G-only (R vs L)	0.19	0.14	0.25	0.74	0.76
G-DED (R vs L)	0.63	0.51	0.07	0.18	0.16
DED-only (R vs L)	0.27	0.5	0.2	0.35	0.35

*P<0.05; **P<0.01; ***P<0.001.G-only Glaucoma group, DED-only dry eye group, G-DED glaucoma with dry eye.

DED-only, dry eye group; G-DED, glaucoma with dry eye; G-only, glaucoma group.

Differences in diversity indices are evaluated using the Wilcoxon test (threshold value of *P*=0.05). Differences between left and right eyes for beta diversity are assessed using an adonis2 test with 1,000 permutations (threshold value of *P*=0.05).

#### Gender-based conjunctival microbiota variation

Differences in diversity indices are evaluated using the t-test (threshold value of *P*=0.05). Differences between the conjunctival microbiota of men and women for beta diversity are assessed using an adonis2 test with 1,000 permutations (threshold value of *P*=0.05).

**Fig. 6.**
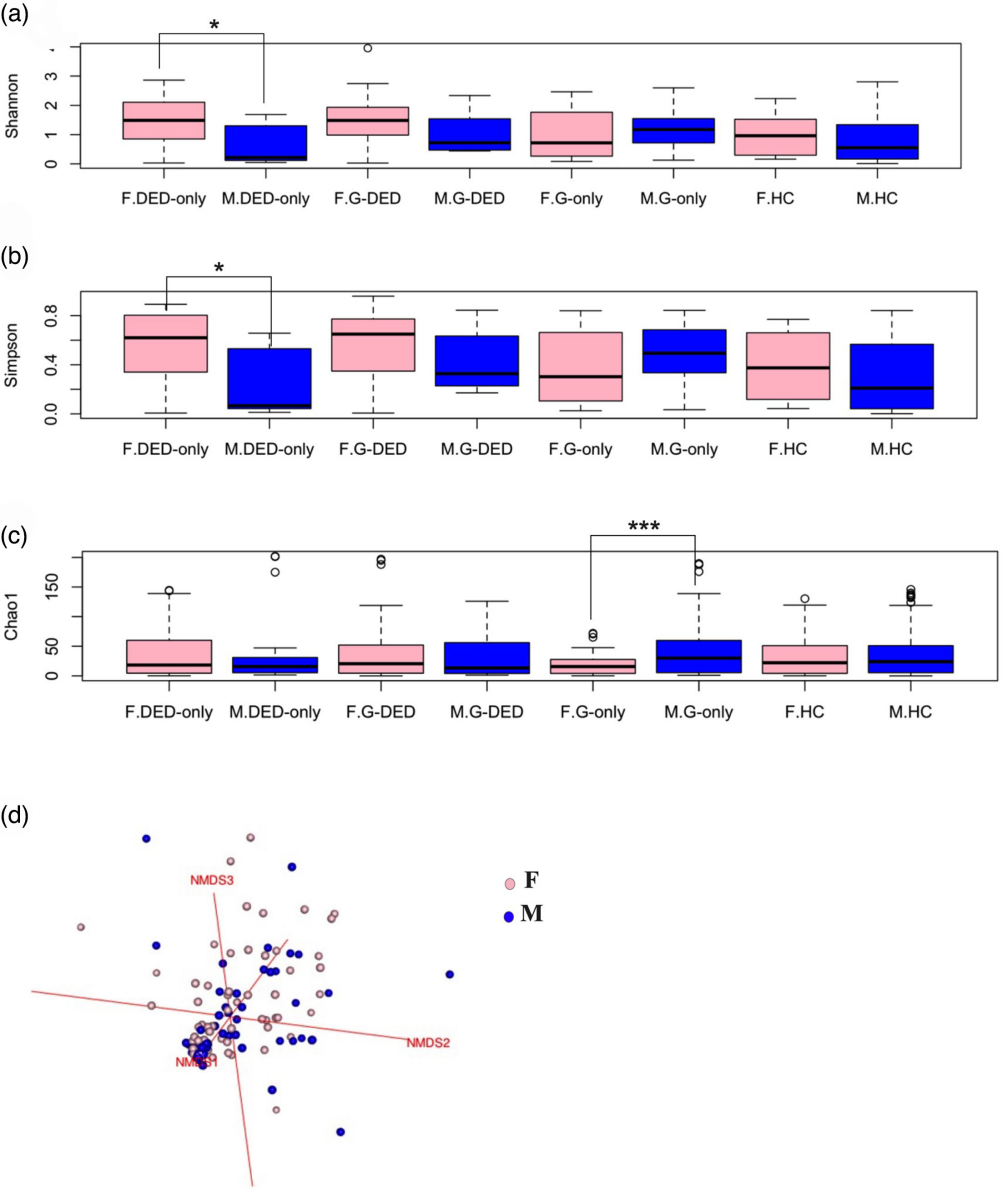
Boxplot visualizing the alpha diversity [based, respectively, on the Shannon index (**a**) and Simpson index (**b**)] and richness [based on the Chao1 index (**c**)], tested by the t-test (threshold value of *P*=0.05). NMDS model (**d**) based on a Bray–Curtis dissimilarity matrix, with data projected into three-dimensional space (*K*=3) and a model stress of 0.1028451, tested by adonis2 (threshold value of *P*=0.05). F, female; M, male.

The t-test showed a significantly higher alpha diversity in women compared with men by both Shannon index (*P<0.05) and Simpson index (*P<0.05) in the DED-only group and by Chao1 index in the G-only group (***P<0.001). However, there was no significant difference in beta diversity between women and men in all groups (*P*>0.05, adonis2) (Table 5, Fig. 6).

**Table 5.** Comparison of alpha diversity and beta diversity of microbiota between women and men by group

Group	Shannon	Simpson	Richness	Bray–Curtis	Jaccard
Comparison	*P*-value	*P*-value	*P*-value	*P*-value	*P*-value
HC M (*n*=38) vs F (*n*=22)	0.59	0.27	0.23	0.199	0.175
G-only M (*n*=19) vs F (*n*=7)	0.73	0.51	0.0003***	0.065	0.053
G-DED M (*n*=6) vs F (*n*=22)	0.241	0.31	0.47	0.77	0.72
DED-only M (*n*=7) vs F(*n*=18)	0.03*	0.037*	0.82	0.33	0.36

*P<0.05; **P<0.01; ***P<0.001.

DED-only, dry eye group; G-DED, glaucoma with dry eye; G-only, glaucoma group.

### Predicted metabolic pathways and functions with PICRUST2

PiCRUST2 was used to predict metagenome functions from the OTU table and raw sequencing reads. Using DESeq2 to compare PiCRUST2 data between the healthy control group and the G-only, G-DED and DED-only groups, we identified differentially abundant features. Specifically, we detected 482, 465 and 194 Enzyme Classification (EC) libraries; 2279, 2268 and 1105 KEGG orthologues; and 58, 34 and 6 MetaCyc pathways, respectively.

Three shared metagenome pathways were differentially abundant in the three pathological groups: ADP-l-glycero-*β*-d-manno-heptose biosynthesis, aerobactin biosynthesis, and guanine 3’-diphosphate 5’-diphosphate (ppGpp) biosynthesis. Eighteen pathways were shared between both glaucoma groups. These pathways encompass essential biosynthetic and catabolic processes such as nt and coenzyme biosynthesis (NAD Biosynthesis II and PWY.1269), LPS biosynthesis critical for bacterial cell walls (KDO.NAGLIPASYN.PWY, PWY.5989 and NAGLIPASYN.PWY), aa degradation (ARGDEG.PWY, ORNARGDEG.PWY and METHGLYUT.PWY), vitamin biosynthesis (BIOTIN.BIOSYNTHESIS.PWY) and fatty acid synthesis (FASYN.INITIAL.PWY). Additionally, they include various other pathways (PWY.6282, PWY.6519, PWY0.862, FUCCAT.PWY, PWYG.321, PWY.7664, PWY0.321 and PWY.5088). These shared pathways indicate critical metabolic activities and microbial functions that may play a role in the ocular surface environment of glaucomatous patients.

## Discussion

Latterly, there has been a growing emphasis on the significance of commensal bacteria within the human body covering microbiota of the oral cavity, gut, vagina and skin. Notably, the OSM is garnering increased attention, in parallel with the emergence of novel methodologies of sequencing. Several studies have acknowledged the crucial role of OSM in maintaining ocular homeostasis [44] and have elucidated its involvement in various ophthalmic diseases, including DED [45,47], conjunctivitis [48], blepharitis [49] and keratitis [50].

Few studies have investigated the relationship between glaucoma and the ocular microbiota [5,6, 29, 30]. As DED occurs at increased prevalence in patients with glaucoma, it is still unclear if the OSM disruptions observed in glaucoma patients are related to their glaucoma or the associated DED. To explore the potential possible differences in OSM for future research on glaucomatous patients with DED, we analysed for the first time the OSM in four groups, the G-only group, the G-DED group, the DED-only group and the control group.

To the best of our knowledge, this is the first study to analyse the OSM of the Tunisian population and broadly corroborate recent research, indicating the predominance of Gram-positive bacteria in the normal OSM [50,51]. Similar to previous reports, we found that *Actinobacteria* (82.21%), *Firmicutes* (11.69%) and *Proteobacteria* (4.74%) were the predominant phyla in the normal OSM [5,27, 50], in contrast to other studies that mentioned the predominance of *Firmicutes* [52,54] or *Proteobacteria* [36,55,59]. At the genus level, the top four genera, *Corynebacterium*, *Staphylococcus, Cutibacterium* and *Streptococcus*, were present in >60% of all samples and may constitute the core OSM, as demonstrated by Kang *et al.* [52]. The high abundance of *Corynebacterium*, *Cutibacterium* and *Staphylococcus* in ocular samples indicates that the conjunctival microbiome of our population exhibits a closer resemblance to the skin flora than any other area of the body [57,60]. Several researchers have identified the presence of *Corynebacterium* as the dominant genus, as reported by [5,61,65]. In our study, this genus was found to be dominant across all groups, present in 100% of samples, and exhibited a high relative abundance (73.59%) in healthy volunteers. It has been established that *Corynebacterium* functions as a commensal on the ocular surface, contributing to the maintenance of normal OSM homeostasis [51]. Furthermore, Pickering *et al.* discovered a positive correlation between the relative abundance of *Corynebacterium* and the expression of mucins (MUC1/MUC4), suggesting its role in the regulation of mucin production [66]. Additionally, it has been noted that the genera *Corynebacterium* and *Streptococcus* play a crucial role in promoting immunity against pathogens such as *Pseudomonas aeruginosa* and *Candida albicans* by facilitating neutrophil recruitment [65]. Kugadas *et al.* highlighted the importance of coagulase-negative *Staphylococcus* (CNS) species, including *Staphylococcus epidermidis*, in regulating neutrophil activation and enhancing resistance to *P. aeruginosa* infection [67]. Furthermore, *Staphylococcus aureus*, a common pathogen causing ocular infections, stimulates pro-inflammatory cytokine secretion and plays a significant role in ocular surface immunity [68]. In addition, other study underlined the role of *Corynebacterium mastitidis* in ocular immune homeostasis and host defence by driving neutrophil recruitment and inducing IL-17 production from conjunctival T cells [69].

In comparison with the healthy control group, the three patient groups exhibited an altered OSM. First, our study had the privilege of quantifying the conjunctival bacterial load using 16S rDNA qPCR, revealing a significant decrease in 16S rDNA copy number in both G-only and DED-only groups (*P*<0.01) in contrast to the control group. This decrease can be explained by the change in the microbial environment due to the antiglaucoma eye drop use and the deficiency of lacrimal film, which may be accompanied by a decrease in nutrients. Second, we detected a significant difference in beta diversity between glaucomatous patients and healthy controls and a significantly higher alpha diversity in the G-DED group in comparison with the control group. This result was confirmed by Lim *et al.*, who noted a higher beta diversity of both eyelid margin microbiota in glaucomatous patients who received topical prostaglandin treatment in contrast to naïf glaucomatous patients [30], and by Chang *et al.*, who found an increase in alpha and beta diversity in both treated and untreated eyes of glaucomatous patients compared to healthy controls.

The very few studies that have investigated the relationship between glaucoma and the OSM have admitted that the use of antiglaucoma eyedrops, containing preservatives, has the potential to alter the conjunctival flora [6,29, 30]. Alternative preservatives in multidose topical ophthalmic formulations play a crucial role in ensuring antimicrobial protection, preserving sterility while cost-effectively extending shelf life. However, their impact extends beyond infectious microbes, as many preservatives can also harm ocular tissues, especially with prolonged exposure [70]. Among them, BAC is the most frequently used and extensively studied preservative in glaucoma eyedrops and artificial tears due to its excellent antibacterial properties and long-term stability. Initially believed to avoid the selection of drug-resistant bacteria when used at realistic concentrations, BAC has been proven to enrich the ocular surface with methicillin-resistant *S. epidermidis* [71]. BAC inhibits the growth of Gram-positive bacteria at lower concentrations, and its potency against Gram-negative bacteria rises at higher concentrations or in the presence of EDTA. BAC can disrupt the tear film, potentially creating a local environment with reduced oxygen levels (hypoxic niche), favouring the growth of Gram-negative anaerobic bacteria [5]. BAC eliminates bacteria through several mechanisms, including enzyme inhibition, albuminous degeneration of the cell membrane and increased membrane permeability. However, the use of these antiseptics at much lower concentrations may lead to bacterial resistance to the antiseptic, as demonstrated by Ohtani *et al.* [29].

Similarly, our study pointed out a significant increase of opportunistic pathogens in glaucomatous patients, especially the Gram-negative bacteria, *Proteobacteria*, including *Bradyrhizobium*, *Escherichia-Shigella*, *Veillonella*, *Pseudomonas* and *Neisseriaceae_ge*, in addition to some *Firmicutes* including *Granulicatella*, *Streptococcus* and* Staphylococcus*. Some bacteria were significantly more abundant in the G-only group compared with the healthy and G-DED groups, namely, *Enterobacter*, *Proteus* and other unclassified *Enterobacteriaceae.* Some other genera were significantly more abundant in the G-DED group, in comparison with the healthy and G-only groups, like *Haemophilus*, *Acinetobacter*, *Paracoccus* and *Kocuria.* Likewise, the previous report mentioned that the eyelid microbiota of glaucomatous patients receiving topical prostaglandin is characterized by a higher abundance of *Azomonas*, *Pseudomonas* and *Granulicatella,* accompanied by a relative decline in the levels of *Delftia* and *Rothia* [30]. Additionally, Chang *et al.* found that the ocular surface of glaucoma patients using IOP-lowering drops with preservatives (BAC) harbours a greater number of anaerobic Gram-negative organisms, including *Akkermansia*, *Faecalibacterium* and *Lachnospiraceae*, that are often detected in the human gut microbiome. Nevertheless, they have reported a decrease in the abundance of Gram-positive bacteria, such as *Corynebacterium* and *Cutibacterium,* in the ocular surface of glaucoma patients [5]. Conversely, we found an increase in the relative abundance of *Cutibacterium* in glaucoma samples, which has been considered a friendly bacteria for the normal ocular surface [72]. Although *Cutibacterium acnes* is considered one of the most dominant commensal bacteria, contributing to ocular surface homeostasis, it also has the potential to act as both an infectious agent, causing late-onset postoperative endophthalmitis and keratitis, and an immunogenic pathogen, resulting in long-lasting inflammation [73]. Therefore, it will be interesting to investigate the characteristics of the *Cutibacterium* strains associated with glaucoma OSM. Additionally, we detected a relative decline, but non-significant, in the abundance of *Corynebacterium* in glaucoma patients*.* Some studies have identified a negative correlation between *Corynebacterium* and other genera [69]. Ocular surface diseases have the potential to disrupt the structure of the ocular surface or markedly elevate the presence of pathogenic bacteria and opportunistic pathogens, potentially resulting in a reduced average relative abundance of *Corynebacterium* [63]. Furthermore, some researchers proved that an elevated prevalence of the *Proteobacteria* could serve as a biomarker of an unstable ocular microbial community, a characteristic similarly observed in gut microbiota [74,75]. Moreover, the significant increase in the prevalence of the *Staphylococcus* genus may indicate the rise of antibiotic-resistant *S. epidermidis*, as observed by Ohtani *et al.*, who reported a higher prevalence of antibiotic-resistant *S. epidermidis* in the conjunctiva of glaucoma patients using antiglaucoma drops preserved with BAC compared with those using drops preserved with a zinc-based ion buffer system [29]. However, another study found that chronic use of topical medications did not affect the aerobic conjunctival bacterial flora of glaucoma patients [76]. These differences between studies may be explained by the microbiota analysis methods used, the different populations studied, the duration of exposure to antiglaucoma eye drops or the presence or absence of DED symptoms.

The most extensively studied ocular disease in relation to OSM is DED. Various researchers have uncovered a robust correlation between the alteration of OSM and the occurrence of DED [76]. Similarly, our study identified a significant difference in beta diversity between individuals with DED and healthy controls. Notable variations in beta diversity between individuals with DED and controls have already been documented [27,31, 32, 34, 38, 47, 77, 78]. However, discordant results have been reported for alpha diversity analysis, varying from higher value in DED [31,34] to lower or similar values like our findings [27,77, 79, 80], likely explained by the greater differences in microbiota composition among individuals [81]. The OSM of the DED patients, in our study, was characterized by a notable increase in Gram-negative bacteria, including *Bradyrhizobium*, *Escherichia-Shigella*, *Veillonella*, *Pseudomonas,* some unclassified *Enterobacteriaceae*, *Acinetobacter* and *Haemophilus.* Additionally, there was an increase in some Gram-positive bacteria such as *Streptococcus*, *Staphylococcus* and *Cutibacterium*. These genera consistently exhibited differential abundance in samples from the dry eye group, regardless of the presence or absence of glaucoma. Dong *et al.* also signalled a higher relative abundance of *Proteobacteria* in individuals with DED [27]. Other researchers revealed a decrease in Gram-positive organisms, such as CNS, *Corynebacterium* and *Streptococcus* [82], accompanied by an increase of Gram-negative organisms from the genera *Pseudomonas*, *Bacteroidetes*, *Bradyrhizobium* and *Bifidobacterium* [34]. Furthermore, *Pseudomonas* has been previously detected on the ocular surface and in individuals with DED [32,65, 77]. In our study, *Acinetobacter* and *Haemophilus* were identified as biomarkers in both DED-only and G-DED groups. However, Gupta *et al.* found a lower level of *Acinetobacter* in patients with dry eye compared to healthy people [36]. Nevertheless, *Acinetobacter* was considered a potential pathogenic bacterial biomarker for Stevens–Johnson syndrome [31], *Demodex* blepharitis [72] and for diabetes [56]. *Haemophilus* was also found on the ocular surface with conjunctivitis, keratitis, blepharitis, dacryocystitis and endophthalmitis [63]. However, Andersson *et al.* signalled that *Haemophilus* was significantly less abundant in dry eye [43].

In our investigation, *Pseudomonas*, *Bradyrhizobium*, *Escherichia-Shigella*, *Streptococcus*, *Veillonella*, *Cutibacterium* and unclassified *Corynebacteriaceae* were detected as bacterial biomarkers in the three pathological groups. However, most of them were previously detected as ocular surface commensals. It seems that the increase in their level may alter the OSM. Moreover, it has been shown that the composition of the tear film can be affected by the OSM because of the use of elements present in tears by various bacteria [83]. For example, the high presence of lipophilic bacteria on the ocular surface could lead to the deterioration of the outermost lipid layer of the tear film, a characteristic indication of evaporative dry eye [81]. So based on this information and the alteration of the OSM in the glaucoma group, the potential increase of some bacteria could be closely related to the dry eye development and may justify the high prevalence of dry eye syndrome in glaucomatous patients, 53.57% in our study.

As observed in our research, the OSM of the G-DED group was the most altered one. It harboured a big number of biomarkers or opportunistic pathogens such as *Paracoccus*, *Exiguobacterium*, *Neisseria*, *Burkholderia*, *Porphyromonas*, *Massilia* and *Serratia.* In the literature, *Paracoccus* was significantly increased in individuals suffering from bacterial keratitis [53] with a positive correlation observed between *Paracoccus* and corneal epithelial injury [84]. Also, *Serratia* has been associated with inflammation and infection related to contact lens use, as well as cases of endophthalmitis following cataract surgery and intravitreal injections [85].

It appears that the G-DED group combined the impacts of DED with the effects of antiglaucoma drops. The discrepancy between the G-DED and DED-only groups lies in the inclusion of BAC in the antiglaucoma eye drops, as both groups utilize cetrimide in some patients. Therefore, the rise in opportunistic pathogens in the G-DED group is likely attributed to the presence of the preservative BAC.

Metagenome inference analysis (PICRUST2) suggests that the differential abundance of certain pathways indicates potential alterations in metabolic activities and microbial community functions on the ocular surface of patients with glaucoma and DED. These changes may either contribute to or result from the disease state. For instance, the high abundance of the ADP-l-glycero-*β*-d-manno-heptose biosynthesis and LPS biosynthesis pathways reflects an increased presence of Gram-negative bacteria. LPS, a major component of the outer membrane of Gram-negative bacteria, is known to upregulate the expression of inflammatory cytokines on the ocular surface. Simmons *et al.* demonstrated that this expression is further increased in dry eyes, contributing to chronic ocular surface inflammation [86]. Similarly, Wang *et al.* reported that increased LPS biosynthesis is associated with elevated levels of inflammatory transcripts, including IL-1*β*, TNF-*α* and CXCL10, in the conjunctiva [87].

The detection of the aerobactin biosynthesis pathway suggests the presence of opportunistic pathogens better equipped to thrive in the altered ocular environment. The ppGpp biosynthesis pathway highlights bacterial adaptation to stress conditions, potentially enhancing bacterial survival and pathogenicity. Additional pathways, such as those involved in aa degradation, vitamin biosynthesis and fatty acid synthesis, reflect significant shifts in bacterial metabolism and interactions with host cells.

**Fig. 7.**
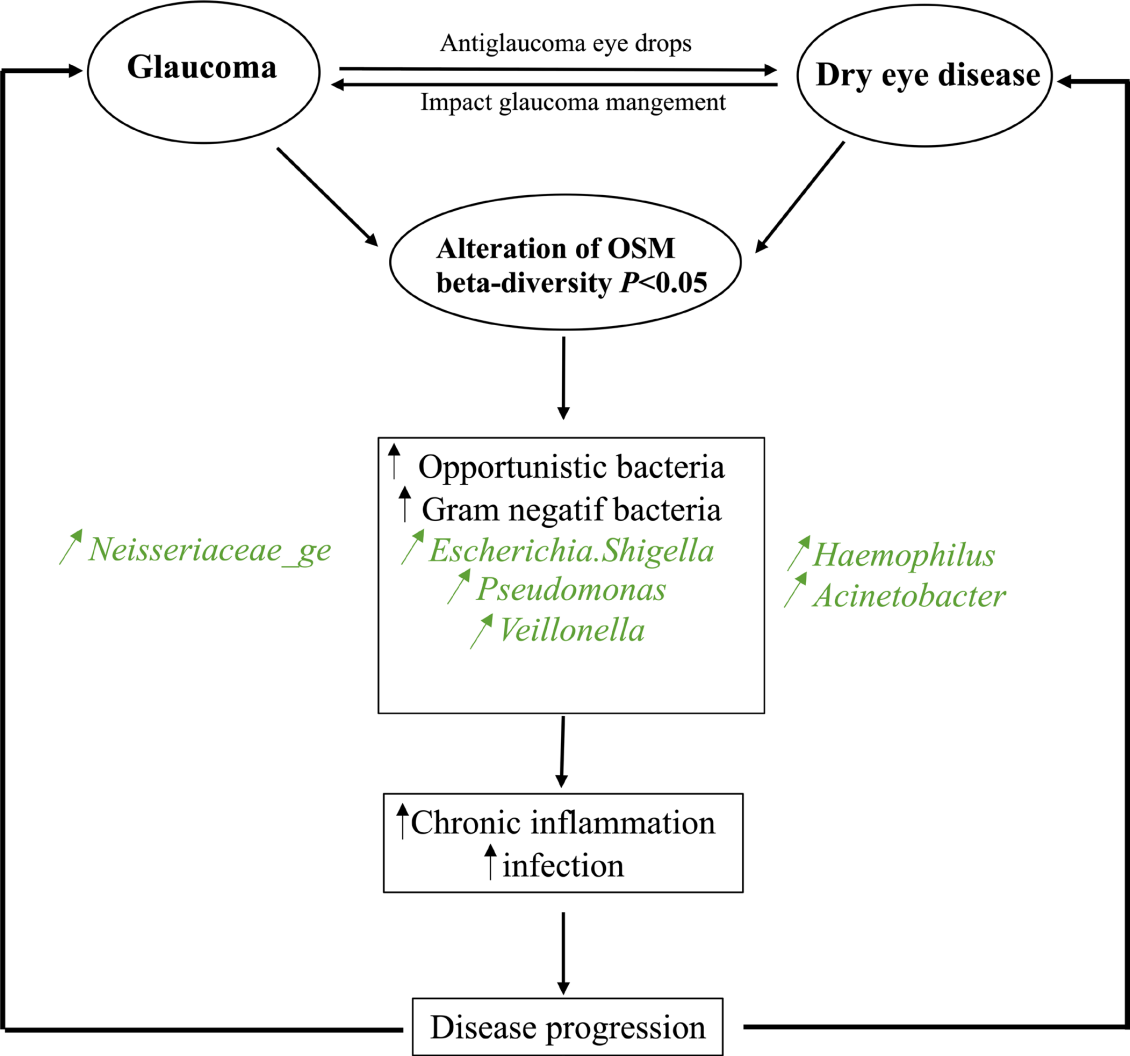
A synthetic representation of the alteration in OSM in patients with glaucoma and DED.

Collectively, these findings suggest a transition in the OSM towards a pro-inflammatory state, which could drive chronic inflammation, ocular surface damage and disease progression in both glaucoma and DED (Fig. 7). These insights into microbial changes may open avenues for innovative therapeutic strategies targeting the OSM to manage disease progression.

The heightened colonization of Gram-negative organisms has previously been linked to a higher incidence of corneal infiltrative events [88] and an elevated risk of infection and inflammation [5].

The variation among studies may be justified by the influence of numerous factors on the OSM, ranging from demographic variables to diverse sampling techniques [89]. Age, sex, diabetes, obesity, race and environmental conditions, sampling site, pressure sampling, and sample sizes of patients, also play a role in impacting the composition of the microbiota [38,50, 57, 90,95]. Despite the effect of diabetes on the alteration of the OSM described in the literature, particularly in association with dry eye syndrome [32,38, 91, 96,102], our results did not show any impact of diabetes on the OSM. These results can be justified by the limited number of diabetic subjects compared to non-diabetic subjects.

Due to the similar environmental pressure, some studies showed that there is no significant difference in the microbiota composition between the right and left eyes [55,95, 103]. Chang *et al.* supposed that the microbiome of the ocular surface in paired eyes might function as a unified microbial ecosystem rather than two distinct systems because of the mechanical transfer of organisms through activities such as eye-rubbing or via the connection between the lacrimal and nasal mucosa, linking the conjunctiva of the left and right eyes [5]. However, other authors found significant differences between the two eyes [104]. In our study, we did not find a significant difference in alpha and beta diversity between left and right eyes by group, but the individual comparison of the two eyes for the same person showed a high dissimilarity score in 59.7% of participants, which is likely related to the highly variable distribution of the OSM [105].

The effect of sex on OSM composition is still debated. Indeed, some authors demonstrated that there is no effect of gender on OSM diversity [50,95]. Ozkan *et al.* in 2017 observed a greater bacterial diversity in males [65]. Additionally, Xu and Zhang reported a higher positive culture rate in male patients compared with female patients [106]. Ozkan *et al.* identified a reduction in richness and diversity in the conjunctival microbiota of females when compared with males [81]. Some studies have indicated that gender impacts only the OSM beta diversity, but not the alpha diversity [95,107]. In our study, there were no significant differences in alpha and beta diversity between males and females in the healthy group. While we detected a significantly higher alpha diversity on the conjunctiva of females compared with males in the DED-only group and a higher richness in males in the G-only group, these results may be justified by the difference in the number of males and females in the two groups.

Our research is subject to several limitations. Firstly, there is a lack of gender matching between groups in our cohort, particularly notable because DED is more prevalent in women. Another limitation of our methodology was the use of the 16S rRNA gene amplicon sequencing approach, which facilitated the identification of bacterial communities within the study population but did not provide information about non-bacterial microbiota and precise classification at the species level, as it would be achievable with metagenomic studies. Furthermore, our study design did not include recruitment of treatment-naïve glaucomatous patients, which limits our ability to identify and analyse the potential effects of treatment on the OSM. Additionally, it is important to acknowledge that the number of patients included in our study, particularly within the pathological groups, was relatively small.

Future research should further include more subjects and include other cofactors like hypertension, origin and other environmental factors.

Looking ahead, the continued advancement of functional metagenomics holds immense potential for reshaping our understanding of OSM dysbiosis and its role in human health.

In conclusion, our study sheds light on the complex interplay between OSM, glaucoma and DED. Through comprehensive analysis using amplicon sequencing, we uncovered diverse bacterial communities and identified significant alterations in microbial composition and diversity among glaucomatous patients with and without DED, as well as DED-only patients, compared with healthy controls. Our findings underscore the importance of considering OSM dysbiosis as a potential contributing factor to the pathogenesis of both glaucoma and DED. The increased presence of opportunistic bacteria in all pathological groups, particularly pronounced in the G-DED group, highlights the potential role of microbial dysbiosis in exacerbating ocular surface inflammation and disease progression. These results affirmed that the alteration in OSM in glaucomatous patients is not due to DED since the OSM of the G-only group was also altered. Overall, our study contributes valuable insights into the microbial profile of glaucoma in patients with DED, expanding our understanding of the OSM and its implications for ocular health and disease management.

## Supplementary material

10.1099/jmm.0.002013 Uncited Supplementary Material 1.

10.1099/jmm.0.002013 Uncited Supplementary Data Sheet 1.

## Abbreviations

ANOVA: analysis of variance
BAC: benzalkonium chloride
CNS: coagulase-negative *Staphylococcus*
DED: dry eye disease
DNA: deoxyribonucleic acid
HC: healthy control
IOP: intraocular pressure
NMDS: non-metric multidimensional scaling model
OSM: ocular surface microbiota
OTU: operational taxonomic unit
PCR: polymerase chain reaction
qPCR: quantitative PCR
rDNA: ribosomal DNA
