# Supplementary material for: Alterations of ocular surface microbiome in glaucoma and its association with dry eye
Source: J Med Microbiol. 2025 May 13;74(5):002013. doi: 10.1099/jmm.0.002013 (PMC12075858; doi:10.1099/jmm.0.002013)
Supplement: Uncited Supplementary Material 1. [file jmm-74-02013-s001.pdf]

Supplementary table 1. Clinical and Demographic Characteristics of Participants (HC, G-Only, G-DED, and DED-Only)

Sample	State	Sex	Age	Diabetes	Hypertension
HC1	HC	F	40	-	-
HC2	HC	F	55	-	-
HC3	HC	M	62	-	+
HC4	HC	F	60	-	-
HC5	HC	F	61	-	-
HC6	HC	M	76	+	-
HC7	HC	F	68	+	-
HC8	HC	M	70	+	+
HC9	HC	F	52	-	-
HC10	HC	M	55	-	+
HC11	HC	M	62	-	-
HC12	HC	M	57	+	-
HC13	HC	M	63	-	-
HC14	HC	M	58	-	-
HC15	HC	F	58	-	+
HC16	HC	F	65	-	+
HC17	HC	M	60	-	-
HC18	HC	F	63	+	+
HC19	HC	F	68	-	-
HC20	HC	M	65	-	-
HC21	HC	M	61	-	-
HC22	HC	M	79	-	+
HC23	HC	M	79	-	+
HC24	HC	M	68	-	-
HC25	HC	M	76	-	-
HC26	HC	M	84	+	+
HC27	HC	M	76	-	-
HC28	HC	M	66	-	-
HC29	HC	M	81	+	-
HC30	HC	F	49	-	-
HC31	HC	F	65	-	+
G-only1	G-only	M	69	-	-
G-only2	G-only	M	84	-	-
G-only3	G-only	M	68	-	-
G-only4	G-only	M	66	-	-
G-only5	G-only	M	82	-	-
G-only6	G-only	M	57	-	-
G-only7	G-only	F	63	+	+
G-only8	G-only	M	67	+	+

G-only9	G-only	F	68	+	+
G-only10	G-only	F	78	-	-
G-only11	G-only	M	69	+	-
G-only12	G-only	M	67	-	-
G-only13	G-only	F	60	-	-
G-DED1	G-DED	F	55	-	-
G-DED2	G-DED	F	62	-	-
G-DED3	G-DED	F	73	-	-
G-DED4	G-DED	M	80	-	-
G-DED5	G-DED	M	75	+	-
G-DED6	G-DED	F	73	-	+
G-DED7	G-DED	F	58	-	-
G-DED8	G-DED	F	47	-	-
G-DED9	G-DED	F	69	+	+
G-DED10	G-DED	F	65	-	-
G-DED11	G-DED	F	52	-	-
G-DED12	G-DED	F	65	-	-
G-DED13	G-DED	M	51	-	-
G-DED14	G-DED	F	63	-	-
G-DED15	G-DED	M	59	+	-
DED-only1	DED-only	F	59	-	-
DED-only2	DED-only	F	44	-	-
DED-only3	DED-only	F	48	-	-
DED-only4	DED-only	M	86	-	-
DED-only5	DED-only	F	40	-	-
DED-only6	DED-only	F	74	-	-
DED-only7	DED-only	F	64	-	-
DED-only8	DED-only	F	63	-	-
DED-only9	DED-only	F	57	-	-
DED-only10	DED-only	M	58	-	-
DED-only11	DED-only	F	62	-	-
DED-only12	DED-only	M	67	+	-
DED-only13	DED-only	M	57	-	-

Abbreviations; HC healthy controls, G-only Glaucoma group, DED-only dry eye group, G-DED glaucoma with dry eye

Supplementary table 2. Most differentially abundant genera between groups, identified by DESeq2.

Phyla	Genus	RA % (G-only)	RA % (HC)	Log2 FC	Padj
Proteobacteria	*Enterobacteriaceae_ge*	5.03	0.21	-9.7	5.7^{e}-18
	Enterobacter	0.44	00	-12.66	4.1^{e}-8
	Veillonella	0.29	0.03	-4.5	0.0029
	Proteus	1.37	00	-12.19	0.004
	Actinomyces	0.84	0.11	-3.8	0.01
	Bradyrhizobium	0.10	00	-7.18	0.01
	Escherichia.Shigella	1.39	0.21	-3.74	0.01
	Neisseriaceae_ge	5.57	0.85	-3.11	0.022
	Pseudomonas	0.46	0.12	-3.08	0.026
Firmicutes	*Streptococcus*	5.25	0.39	-4.34	3.5^{e}-5
	Granulicatella	0.60	0.03	-4.98	0.006
	Gemella	0.24	0.02	-3.93	0.022
Actinobacteriota	*Cutibacterium*	10.19	6.60	-2.22	0.014
	Corynebacteriaceae_ge	0.20	0.09	-2.05	0.03
Fusobacteriota	*Fusobacterium*	0.13	0.02	-4.37	0.022
**Phyla**	**Genus**	**RA % (G-DED)**	**RA % (HC)**	**Log2 FC**	**Padj**
Proteobacteria	*Haemophilus*	4.03	0.03	-6.96	3.21^{e}-8
	Escherichia.Shigella	1.45	0.21	-6.5	4.18^{e}-8
	Acinetobacter	0.77	0.11	-6.18	5.6^{e}-8
	Burkholderia	0.16	00	-25.4	7.41^{e}-22
	Neisseria	0.14	0.04	-6.06	6.8^{e}-7
	Pseudomonas	0.52	0.12	-5.38	1.9^{e}-6
	Veillonella	0.26	0.03	-5.24	4.5^{e}-5
	Enterobacterales_ge	0.32	0.02	3.26	0.007
	Paracoccus	0.87	0.47	-2.86	0.007
	Neisseriaceae_ge	424	0.85	-3.11	0.008
	Serratia	3.10	00	-6.17	0.015
	Rhodobacteraceae_ge	0.14	0.04	-3.11	0.02
	Massilia	0.19	0.03	-3.52	0.032
Firmicutes	*Staphylococcus*	14.14	7.07	-4.62	4.56^{e}-13
	Exiguobacterium	2.84	0.04	-7.99	1.7 ^{e}-7
	Streptococcus	5.27	0.39	-4.47	2.8^{e}-6
	Granulicatella	0.08	0.03	-4.03	0.012
Actinobacteriota	*Cutibacterium*	9.56	6.60	-3.94	0.0006
	Corynebacteriaceae_ge	0.39	0.09	-3.31	1.65^{e}-5
	Kocuria	0.56	0.31	-2.94	0.028
Fusobacteriota	*Leptotrichia*	0.16	0.04	-4.15	0.032
Bacteroidota	*Prevotella_7*	0.11	0.01	-8.14	5.34^{e}-8
	Prevotella	0.09	00	-9.36	4.4^{e}-6
	Porphyromonas	0.12	0.03	-3.6	0.042
**Phyla**	**Genus**	**RA % (DED-only)**	**RA % (HC)**	**Log2 FC**	**Padj**
Proteobacteria	*Bradyrhizobium*	1.41	00	-14.13	1.11^{e}-9
	Escherichia.Shigella	2.29	0.21	-3.09	0.026
	Haemophilus	0.19	0.03	-3.36	0.02
	Acinetobacter	1.38	0.11	-2.65	0.048
	Enterobacteriaceae_ge	0.40	0.21	-3.59	0.0053
	Pseudomonadaceae_ge	0.08	0.20	-4.14	0.0053
	Pseudomonas	0.97	0.12	-4.66	0.0002
	Veillonella	0.63	0.03	-3.55	0.015
Firmicutes	*Staphylococcus*	12.55	7.07	-4.14	0.0027
	Streptococcus	1.90	0.39	-3.51	0.0008
Actinobacteriota	*Cutibacterium*	8.74	6.60	-2.11	0.013
	Corynebacteriaceae_ge	0.11	0.09	-1.84	0.040
**Phyla**	**Genus**	**RA % (G-DED)**	**RA % (G-only)**	**Log2 FC**	**Padj**
Proteobacteria	*Haemophilus*	4.03	0.11	-4.52	0.0085
	Enterobacteriaceae_ge	0.15	5.03	4.41	0.0056
	Acinetobacter	0.77	0.04	-5.79	0.00016
	Proteus	0.01	1.37	11.95	0.015
	Rhodobacteraceae_ge	0.14	00	-4.61	0.0085
	Enterobacter	0.02	0.44	6.15	0.044

	Paracoccus	0.87	0.21	-3.11	0.026
Firmicutes	*Staphylococcus*	14 .14	08.59	-3.11	0.0003
Actinobacteriota	*Micrococcus*	0 .09	0.41	-4.81	0.026
	Kocuria	0.56	0.13	-3.5	0.044
**Phyla**	**Genus**	**RA % (G-only)**	**RA % (DED-only)**	**Log2 FC**	**Padj**
Proteobacteria	*Enterobacteriaceae_ge*	5.03	0.40	-6.10	0.0008
	Enterobacter	0.44	00	-9.67	0.029

*p<0.05; **p<0.01; ***p<0.001. Abbreviations; RA Relative Abundance, Log2 FC Logarithme base 2 du Fold Change**,** Padj Adjusted p-value.
